# Expanding the Spectrum of Uropathogens: A Rare Human Urinary Tract Infection by Rodentibacter pneumotropicus

**DOI:** 10.7759/cureus.103004

**Published:** 2026-02-05

**Authors:** Harish Prabhu, Madhuri Harshan

**Affiliations:** 1 Nephrology, Burjeel Royal Hospital, Al Ain, ARE; 2 Internal Medicine, Independent Research, Al Ain, ARE

**Keywords:** pasteurella pneumotropica, recurrent urinary tract infection, rodentibacter, rodents, urinary tract infections

## Abstract

*Pasteurella pneumotropica* (now reclassified as *Rodentibacter pneumotropicus*) is a gram-negative coccobacillus primarily associated with rodents, with human infections being exceedingly rare. We report an unusual case of urinary tract infection (UTI) caused by *P. pneumotropica* in a 56-year-old man with multiple comorbidities, including chronic kidney disease (CKD), type 2 diabetes mellitus, systemic hypertension, and coronary artery disease (status post pacemaker insertion), who experienced multiple episodes of urinary tract Infection over a period of two years. His latest urine culture revealed growth of *Pasteurella pneumotropica*, a rare zoonotic gram-negative organism not commonly implicated in human disease. This case contributes to expanding the current understanding of human infections caused by *Rodentibacter pneumotropicus*, emphasizing the need for clinical awareness, microbiological vigilance, and careful antimicrobial stewardship.

## Introduction

*Pasteurella* species are nonmotile, gram-negative rods or coccobacilli that primarily affect laboratory mice and rats [1]. Human infections are extremely rare, with only a few cases reported worldwide [2,3]. Risk factors for *Pasteurella* infection include animal exposure (especially cat or dog bites/scratches or close contact) and, particularly in the elderly, immunocompromised status, urinary tract abnormalities, or catheter use. We present a rare case of a urinary tract infection caused by *Pasteurella pneumotropica* in a male patient with chronic kidney disease (CKD), which has been described only once previously in the medical literature [4].

## Case presentation

A 56-year-old male electrician by profession, with a medical history of type 2 diabetes mellitus, systemic hypertension, coronary artery disease (status post pacemaker insertion), and stage 3 chronic kidney disease (CKD), first presented in August 2023 with complaints of dysuria and foul-smelling urine.

**Figure 1:**
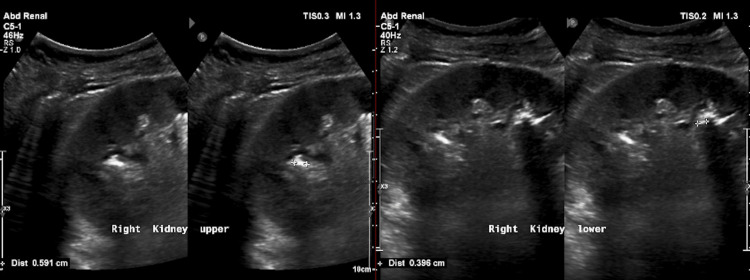
Ultrasound of the abdomen showing renal calculi in the right kidney measuring 5.9 mm

Initial evaluation revealed elevated serum creatinine at 148 µmol/L and an estimated glomerular filtration rate (eGFR) of 64 mL/min/1.73 m^2^. Urinalysis showed significant pyuria, and urine culture grew *Klebsiella pneumoniae*. He was treated with culture-sensitive antibiotics. Given the presentation of UTI in a male patient, an ultrasound of the abdomen and pelvis was performed, which showed normal-sized kidneys with preserved echogenicity and corticomedullary differentiation, along with two non-obstructive renal calculi in the right kidney, measuring 5.9 mm in the upper calyx and 3.9 mm in the lower calyx on February 4, 2024 (Figure 1).

There was no prostatic tenderness or evidence of prostatic abscess on clinical examination. His diabetes was well-controlled, with a hemoglobin A1C (HbA1c) of 5.4%, and his hemoglobin level was 13.4 g/dL. As the stones were non-obstructive, he was advised conservative medical management, including adequate hydration and a low-salt, low-protein diet.

Two months later, he experienced another UTI, presenting with similar symptoms. Urine culture this time grew *Klebsiella ozaenae*, for which he was again treated with appropriate antibiotics based on sensitivity.

Following this episode, the patient remained asymptomatic for eight months and continued on regular follow-up. However, in May 2024, he had another UTI episode, followed by three more episodes in the same year. He received appropriate antibiotic treatment each time.

**Figure 2:**
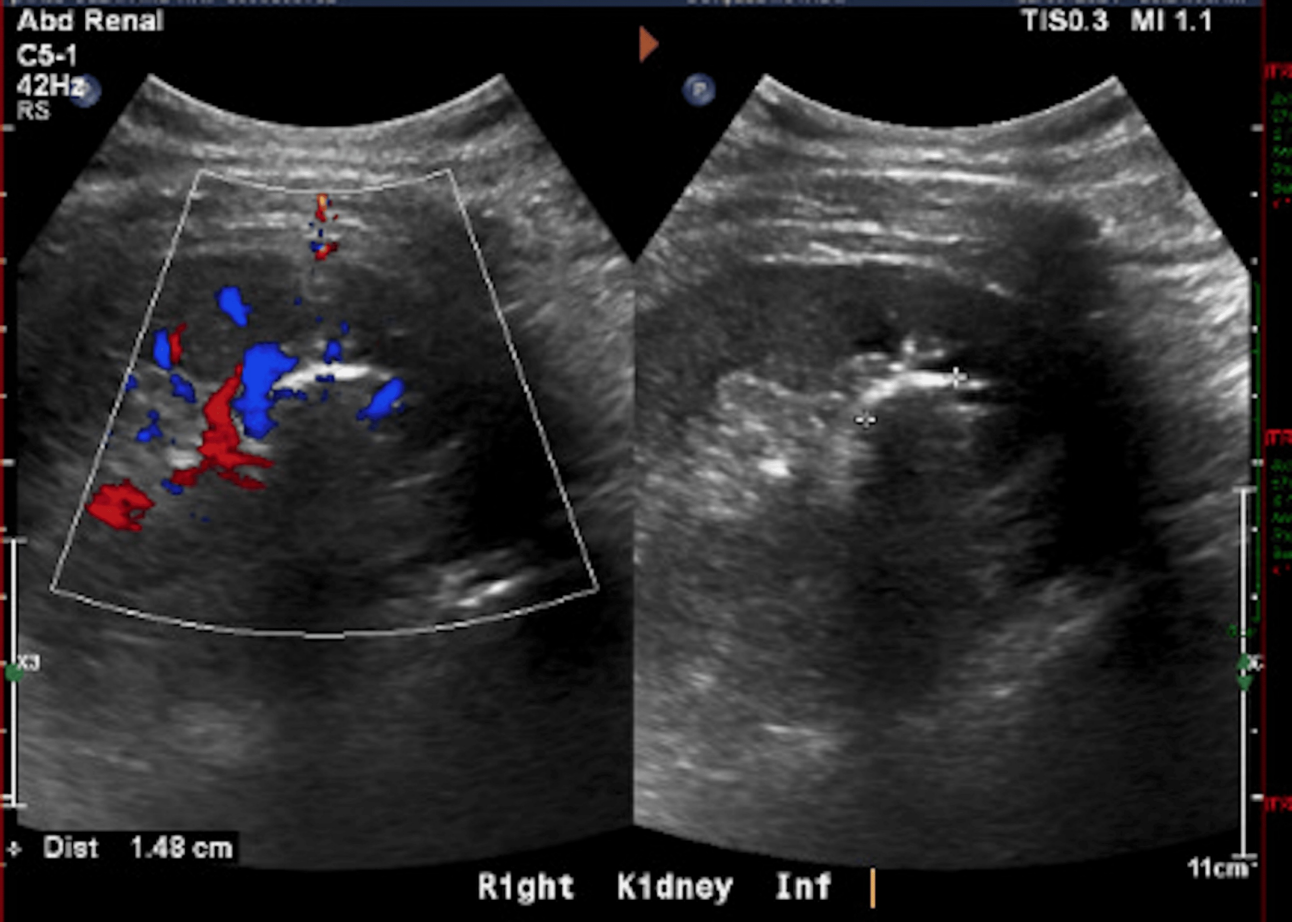
Repeat ultrasound of the right kidney showing a renal calculus measuring 1.4 cm

A repeat ultrasound showed kidneys of normal size and echogenicity; however, the right renal calculus had increased in size to 1.4 cm on June 4, 2024 (Figure 2). Due to the recurrent infections and increase in stone size, urological intervention was advised for stone removal, but the patient declined the procedure.

Given the recurrent nature of his UTIs, he was started on weekly prophylactic fosfomycin, after which he remained infection-free for six months.

In August 2025, the patient again presented with symptoms of UTI. This time, urine culture revealed *Pasteurella pneumotropica*, a rare gram-negative organism. The isolate was sensitive to ceftriaxone, and he was treated successfully with intravenous ceftriaxone for a total of seven days.

## Discussion

Recurrent urinary tract infection (UTI) is defined as ≥2 episodes of UTI within six months or ≥3 episodes within a year. It predominantly affects women, with an incidence of 20%-30%, while in men under 50 years, the incidence drops to <1% and rises to 5%-10% in those over 50 years [5].

Risk factors for recurrent UTI differ between genders. In men, common risk factors include benign prostatic hyperplasia (BPH), urinary catheterization, urinary calculi, diabetes mellitus, chronic kidney disease, immunosuppression, history of recurrent UTIs, and structural urological abnormalities [6].

Recurrent UTIs in men are often polymicrobial and more likely to involve multidrug-resistant organisms (MDROs). The common causative organisms include *Escherichia coli*, *Klebsiella pneumoniae*, *Proteus mirabilis*, *Pseudomonas aeruginosa*, and *Enterococcus* spp., with *E. coli* being the most frequent pathogen [7-9].

Our patient was at high risk for recurrent UTI due to diabetes mellitus, CKD, and the presence of renal calculi. His initial infections involved typical uropathogens (*K. pneumoniae* and *K. ozaenae*), and he responded to culture-directed antibiotics. Despite prophylactic therapy, in August 2025, he developed another UTI caused by a rare gram-negative organism, *Pasteurella pneumotropica*.

*Pasteurella pneumotropica* is a nonmotile, gram-negative rod or coccobacillus, typically found in laboratory rodents and also common in wild rodents outside laboratory settings. Human infections are extremely rare, with only a handful of cases documented globally, all primarily in immunocompromised individuals. Reported infections include septicemia, osteomyelitis, septic arthritis, and soft tissue infections, but urinary tract infections caused by this organism are exceptionally rare [10].

To date, only one case of UTI caused by *P. pneumotropica* has been documented, reported by Soto-Vega et al. in 2023 [4]. That case involved a 37-year-old immunocompetent man with persistent fever and leukocytosis, who underwent transurethral resection of the prostate and was treated with fluoroquinolones [4]. In contrast, our patient had a history of recurrent UTIs and presented with persistent dysuria, but no systemic symptoms. He required treatment with intravenous ceftriaxone, as the organism was resistant to most oral antibiotics.

Importantly, in 2017, *P. pneumotropica* was reclassified under a new genus, *Rodentibacter pneumotropicus*, after molecular analysis (16S rRNA sequencing, multilocus sequence analysis, and whole-genome comparisons) revealed that it did not phylogenetically cluster within the *Pasteurella* genus [11]. These bacteria are commensals in rodents but can act as opportunistic pathogens, especially in immunocompromised or stressed animals. In rodents, infections can manifest as conjunctivitis, rhinitis, pneumonia, otitis media, or mastitis [12-15].

There are no specific treatment guidelines for *Pasteurella pneumotropica. *They are mostly sensitive to penicillin, cephalosporins, and fluoroquinolones. Notably,* Pasteurella* is resistant to macrolides. Treatment duration is based on severity, with mild infections requiring 5-7 days and severe infections needing 14 days.

Notably, our patient had no known exposure to rodents or laboratory animals, which raises the possibility of environmental acquisition or an undetected exposure. The isolate was not susceptible to oral antibiotics, reinforcing concerns about emerging resistance among atypical pathogens.

This case highlights the expanding microbial spectrum in UTIs, especially in at-risk populations. It also underscores the importance of culture-directed therapy, particularly in recurrent or atypical infections, avoiding empirical overuse of antibiotics, which can promote resistance, considering rare organisms when patients fail conventional treatment, and reporting such cases to improve awareness and inform diagnostic vigilance.

## Conclusions

This case report highlights the expanding spectrum of organisms responsible for urinary tract infections. We present a rare case of urinary tract infection caused by *Rodentibacter pneumotropica* (formerly *Pasteurella pneumotropicus*).

This case highlights the importance of clinicians remaining vigilant for atypical pathogens, particularly in patients with persistent or recurrent urinary symptoms and underlying comorbidities, such as chronic kidney disease and renal calculi. Early identification and targeted antimicrobial therapy are crucial for preventing complications and improving outcomes in these uncommon presentations.
